# Anesthetic management of a patient with factor VII deficiency undergoing laparoscopic colectomy: a case report

**DOI:** 10.1186/s40981-016-0059-0

**Published:** 2016-10-19

**Authors:** Akari Yoshida, Yoshiki Kimoto, Kanako Ejiri, Yasuyuki Mitani, Tomoyuki Kawamata

**Affiliations:** 1 Department of Anesthesiology, Wakayama Medical University, 811-1 Kimiidera, Wakayama City, 641-0012 Japan; 2 Second Department of Surgery, Wakayama Medical University, 811-1 Kimiidera, Wakayama City, 641-0012 Japan

**Keywords:** Congenital FVII deficiency, Coagulation management, Recombinant activated FVII, Surgical bleeding

## Abstract

**Background:**

Congenital factor VII (FVII) deficiency is a rare autosomal recessive coagulation disorder that is characterized by prolongation of prothrombin time. Recombinant activated FVII (rFVIIa) is widely used in the management of bleeding in patients with congenital FVII deficiency. We experienced anesthetic management of a patient with congenital FVII deficiency who was scheduled for laparoscopic colectomy using rFVIIa.

**Case presentation:**

We report a 67-year-old man with rectal cancer who was diagnosed with congenital FVII deficiency. He was scheduled for laparoscopic colectomy. General anesthesia was performed with propofol, remifentanil, and rocuronium without epidural anesthesia. For coagulation management, 1 mg of rFVIIa was intravenously administered before starting surgery. During surgery, FVII activity and prothrombin time-international normalized ratio (PT-INR) were maintained to be above 10 % and within the normal range (0.8–1.2), respectively. The surgery was uneventfully completed.

**Conclusions:**

We reported successful management of a patient with congenital FVII deficiency undergoing laparoscopic colectomy with monitoring of FVII activity and/or PT-INR.

## Background

Factor VII (FVII), which is a vitamin K-dependent coagulation factor, plays a role in initiation of the extrinsic coagulation pathway with tissue factor. Its congenital deficiency is a rare autosomal recessive coagulation disorder with an estimated prevalence of about 1:500,000 [1]. FVII deficiency can be suspected when a coagulation screening test reveals an isolated prolongation of prothrombin time (PT) with a normal activated partial thromboplastin time (aPTT). Congenital FVII deficiency is diagnosed when patients show a decrease in only FVII among the coagulant factors without having any liver disease, not taking any medication that inhibits FVII activity, and not having an acquired inhibitor of FVII. Patients with congenital deficiency of FVII have an increased risk of postoperative bleeding [2]. Administration of recombinant activated FVII (rFVIIa) is widely accepted as a therapeutic option for both spontaneous bleeding and surgical bleeding in patients with congenital FVII deficiency. However, a treatment strategy for surgical bleeding has not been established [3], and there is only one report regarding anesthetic management of a patient with factor VII deficiency [4]. Here, we report anesthetic management of a patient with congenital FVII deficiency who was scheduled for laparoscopic colectomy. We intravenously administered rFVIIa and monitored FVII activity and/or prothrombin time-international normalized ratio (PT-INR) during the perioperative period.

## Case presentation

Written informed consent was obtained from the patient for this case report.

A 67-year-old man (170 cm, 71 kg) was admitted to our hospital to undergo laparoscopic colectomy for rectal cancer. He had no significant past medical history including bleeding disorder, and his family history was also unremarkable. Preoperative laboratory tests showed an abnormal coagulation profile. Although his aPTT and platelet count were normal (29.6 s and 23.1 × 10^4^ μl, respectively), his PT was prolonged. PT was 43.4 s and PT-INR was 3.58 (normal range, 0.8–1.2). He did not suffer from liver disease, and laboratory data for liver function were normal. Given his high PT-INR with normal aPTT, FVII deficiency was suspected. A following laboratory test of coagulation factors showed that only FVII activity was significantly decreased to 1.4 % of normal. Since he was not taking any drugs that inhibit FVII activity and had no FVII inhibitor, he was diagnosed with congenital FVII deficiency.

We planned anesthetic management by general anesthesia without epidural anesthesia for his surgery to avoid bleeding associated with an epidural block procedure. Fresh frozen plasma (FFP) or rFVIIa was not transfused preoperatively. In the operating room, an electrocardiogram, noninvasive blood pressure, percutaneous oxygen saturation, and nasopharyngeal temperature were monitored. Radial artery pressure was also monitored. General anesthesia was induced with propofol (3.0 μg ml^−1^ via target-controlled infusion (TCI)) and remifentanil (0.3 μg kg^−1^ min^−1^), and then, the trachea was intubated following administration of rocuronium. Anesthesia was maintained with propofol (2.0–3.0 μg ml^−1^ via TCI), remifentanil (0.1–0.3 μg kg^−1^ min^−1^), rocuronium (5.0–7.0 μg kg^−1^ min^−1^), and oxygen-in-air gas mixture.

During the perioperative period, FVII activity and PT-INR were monitored. PT-INR and FVII activity during the clinical course are summarized in Table 1. Since FVII activity and PT-INR were 1.5 % of normal and 3.66, respectively, before starting surgery, 1 mg of rFVIIa (15 μg kg^−1^), which is recommended by the manufacturer for patients with congenital FVII deficiency, was intravenously administered immediately prior to surgery. Then, surgery was started. It has been suggested that FVII activity of less than 10 % is associated with bleeding complications [5], and since the half-life of rFVII is 2–4 h, we checked FVII activity during surgery every 2 h to maintain FVII activity above 10 %. Two hours after administration of rFVIIa, FVII activity was 205.7 % of normal, and PT-INR was normalized to 0.9. The surgery was uneventfully completed in 3 h and 46 min. Since FVII activity and PT-INR were 86.8 % and 0.89, respectively, after completion of surgery (4 h after administration of rFVIIa), additional rFVIIa or FFP was not transfused. Blood loss was 15 ml, and bleeding/oozing was not observed. Since hemoglobin at the end of the surgery was 12.1 g/dL, blood was also not transfused. After the surgery, fentanyl-based intravenous patient-controlled analgesia (iv-PCA) was started for postoperative pain management. The iv-PCA regimen was as follows: a solution of 1400 μg fentanyl and 2.5 mg droperidol mixed with physiological saline to a total volume of 100 ml, 1 ml of bolus dose, 2 ml h^−1^ of basal infusion, and 15 min of a lockout time. The patient was transferred to the general ward in a stable condition without any sign of hemorrhage.

**Table 1:** PT-INR and FVII activity during perioperative period

	Preoperative examination	During anesthesia			POD 1	POD 3	POD 4
		Before rFVIIa	2 h after rFVIIa	4 h after rFVIIa			
PT-INR	3.58	3.66	0.9	0.89	2.18	2.93	1.46
FVII activity (%)	1.4	1.5	205.7	86.8	NA	1.7	NA

*PT-INR* prothrombin time-international normalized ratio, *FVII* factor VII, *POD* postoperative day, *rFVIIa* recombinant activated FVII, *NA* not assessed

The amount of drainage from the pelvic tube was 50 ml on postoperative day (POD) 1, but it increased to 125 ml and became bloody on POD 2. Therefore, 1 mg of rFVIIa was administered intravenously on POD 2. However, FVII activity and PT-INR were 1.7 % of normal and 2.93, respectively, and the fluid from the pelvic tube was still bloody on POD 3. Therefore, rFVIIa was repeatedly administered every 8 h until POD 4. Since the amount of drainage from the pelvic tube was decreased to 55 ml and the drainage had become serous fluid on POD 4, the pelvic drainage tube was removed on POD 4. His postoperative pain was controlled well by iv-PCA, and the pain intensity score at rest was 0–2 of 10 in numeric rating scale through the postoperative period. The patient was discharged without any episode of bleeding or thrombosis on POD 10.

### Discussion

Although congenital deficiency of FVII increases the risk of posttraumatic and postoperative bleeding [2], it is difficult to predict the perioperative risk of bleeding due to a poor correlation between FVII activity and severity of bleeding in patients with congenital FVII deficiency [6]. A recent retrospective study has suggested that FVII activity of less than 10 % is a risk factor for bleeding complications associated with a surgical procedure [5]. In our case, since preoperative FVII activity and PT-INR were 1.4 % and 3.58, respectively, we planned to substitute FVII.

Prothrombin complex concentrates, FFP, and rFVIIa have been used to increase plasma FVII level. Administration of rFVIIa has become the most widely accepted therapeutic option for congenital FVII deficiency in surgical settings because no human serum or proteins are used in the production of rFVIIa [7]. National guidelines regarding the use of blood products in Japan recommend administration of each coagulation factor concentrate rather than FFP if it is available. There are several reports showing the effectiveness of administration of rFVIIa for perioperative coagulation management of a patient with congenital FVII deficiency [3, 8].

While the optimal regimen for perioperative administration of rFVIIa has still not been established, the recommended dose range of rFVIIa for general surgery is 15–30 μg kg^−1^ every 4–6 h until hemostasis is achieved [9]. At least 10 % of FVII activity would be needed for hemostasis [5]. In our case, the decision to administer rFVIIa was made on the basis of assessment of FVII activity and PT-INR. Accordingly, bolus administration of 15 μg kg^−1^ of rFVIIa was sufficient to maintain FVII activity above 10 %, and additional administration of rFVIIa was not needed during surgery. Since the time course of FVII activity after rFVIIa administration is different in each patient [2], the measurement of FVII activity would be useful for the determination of the dose and timing of rFVIIa administration for each patient.

Possible complications of replacement therapy with rFVIIa are thrombosis and production of antibodies against FVII [9, 10]. A previous systematic review showed that rFVIIa dose-dependently increased the risk of arterial thromboembolic events in patients who received a placebo or rFVIIa at doses of less than 80 μg kg^−1^, 80 to 120 μg kg^−1^, or more than 120 μg kg^−1^ [11]. Since the dose we administered (15 μg kg^−1^), which is recommended for congenital FVII deficiency, is lower than the doses used in that study, the risk for thromboembolic events in our case might have been relatively low. However, since surgical stress is a well-known risk factor for thrombosis, administration of rFVIIa during surgery may increase the incidence of thromboembolic events. The measurement of the FVII activity and PT-INR may be helpful for preventing overdose administration. Recently, continuous infusion of rFVIIa has been used to maintain the appropriate plasma concentration of FVII during surgery [12]. It has been shown that continuous infusion is safe, effective, well-tolerated, and cost-effective compared to bolus administration. However, bolus administration rather than continuous infusion is recommended in Japan.

## Conclusions

We reported successful management of a patient with congenital FVII deficiency undergoing laparoscopic colectomy with monitoring of FVII activity and/or PT-INR.

## Abbreviations

aPTT: Activated partial thromboplastin time
FFP: Fresh frozen plasma
FVII: Factor VII
iv-PCA: Intravenous patient-controlled analgesia
POD: Postoperative day
PT: Prothrombin time
PT-INR: Prothrombin time-international normalized ratio
rFVIIa: Recombinant activated FVII
TCI: Target-controlled infusion
